# Acute pancreatitis temporally associated with COVID-19 pneumonia in a patient with post-tuberculosis chronic pulmonary aspergillosis: a case report

**DOI:** 10.3389/fmed.2026.1828229

**Published:** 2026-06-09

**Authors:** Wenqing Yu, Peiqing Yang, Mingdong Ding, Li Guo, Ying Liu, Daming Zhou, Chengjing Gu

**Affiliations:** 1 Department of Infectious Diseases, Taizhou School of Clinical Medicine, The Affiliated Taizhou People’s Hospital of Nanjing Medical University, Nanjing Medical University, Taizhou, Jiangsu, China; 2 Department of Pharmacy, Taizhou School of Clinical Medicine, The Affiliated Taizhou People’s Hospital of Nanjing Medical University, Nanjing Medical University, Taizhou, Jiangsu, China

**Keywords:** acute pancreatitis, COVID-19, Pneumonia, post-tuberculosis lung disease, pulmonary aspergillosis

## Abstract

This report describes a 59-year-old woman with a history of malignancy and post-tuberculosis lung disease complicated by chronic cavitary pulmonary aspergillosis. She was admitted with worsening hemoptysis and underwent bronchial artery embolization. However, she subsequently developed massive post-procedural hemoptysis, requiring mechanical ventilation. Sputum metagenomic next-generation sequencing detected SARS-CoV-2 and bacterial pathogens, prompting Paxlovid treatment for COVID-19 pneumonia. While her respiratory symptoms improved, epigastric pain developed. Based on elevated serum amylase/lipase and CT-confirmed peripancreatic inflammation, she was diagnosed with acute pancreatitis. One year later, pulmonary tuberculosis and liver metastasis recurred. This case highlights acute pancreatitis temporally associated with COVID-19 pneumonia in a patient with multiple competing risk factors. Further, this case underscores the diagnostic complexity of structural lung disease with overlapping infections such as COVID-19 and stresses on the need for long-term surveillance.

## 1 Introduction

Coronavirus Disease 2019 (COVID-19) infection, caused by SARS-CoV-2, primarily manifests as respiratory distress but is significantly associated with extrapulmonary complications such as pancreatic involvement (1). Acute pancreatitis (AP), most commonly caused by gallstones and alcohol, may also be potentially associated with SARS-CoV-2, with proposed mechanisms including direct viral injury, systemic inflammation, or microvascular thrombosis (2, 3). However, the causal relationship between SARS-CoV-2 infection and acute pancreatitis remains controversial (4). We report a case of AP that developed during the course of COVID-19 pneumonia in a patient with a notably intricate medical history, including malignant gastrointestinal stromal tumor (GIST), post-tuberculosis lung disease (PTLD), and chronic cavitary pulmonary aspergillosis (CCPA), highlighting the diagnostic and therapeutic challenges.

## 2 Case report

The detailed sequence of clinical events, from admission to the 1-year follow-up, is summarized in Table 1. A 59-year-old woman presented with hemoptysis that had worsened over the last 4 days. She denied having fever, sputum, chest pain, or dyspnea. She had a 1-year history of hemoptysis. In addition, her past history included an adrenal cell tumor surgery in 2006 and a radical pancreaticoduodenectomy for GIST in 2017 that was complicated by biliary fistula, for which she had received more than 5 years of targeted therapy (imatinib followed by regorafenib). She had smear-positive pulmonary tuberculosis (2020) for which she been undergoing irregular treatment due to drug-induced liver injury. In addition, recurrent severe hemoptysis necessitated four arterial embolizations. CCPA was clinically diagnosed, and she received voriconazole for 2 months.

**TABLE 1:** Timeline of clinical events and management.

Time point	Clinical events and interventions
Day 1	Admission for worsening hemoptysis; initial laboratory investigations and imaging; initiation of oxygen therapy, voriconazole, and broad-spectrum antibiotics.
Day 8	Persistent hemoptysis; fifth bronchial artery embolization performed.
Day 9	Massive post-procedural hemoptysis and suffocation; immediate intubation and initiation of invasive mechanical ventilation.
Day 12	Successful extubation; sputum metagenomic next-generation sequencing (mNGS) returns positive for SARS-CoV-2, *Escherichia coli*, and *Enterococcus faecium*.
Day 13	Initiation of oral Paxlovid (5-day course) and intravenous dexamethasone (tapering course) for COVID-19 pneumonia and systemic inflammation.
Day 18	Resolution of fever and improved respiratory status; onset of acute epigastric tenderness.
Day 19	Diagnosis of acute pancreatitis based on elevated amylase/lipase (>3x ULN) and CT findings; initiation of fasting, octreotide infusion, and fluid resuscitation.
Day 20–31	Stabilization of pancreatic enzyme levels; transition from parenteral to enteral nutrition via nasojejunal tube.
Day 32	Discharge from the hospital.
12-month follow-up	Recurrence of pulmonary tuberculosis (confirmed via GeneXpert) and GIST liver metastasis; initiation of TB therapy; absence of late pancreatic complications.

On admission (Day 1), the following observations were made: body temperature: 37.2 °C; pulse: 108 beats/min; BP: 127/91 mmHg; respiratory rate: 20 breaths/min; and oxygen saturation: 96% on room air. She had an anemic appearance, with a little worse in spirit. The left upper lung showed diminished breath sounds without crackles or wheezes. A surgical scar was seen in the middle of the upper abdomen. The abdomen was flat and soft, without tenderness or rebound pain.

Initial laboratory investigations revealed the following findings: hemoglobin level, 88 g/L; WBC count, 7.83 × 10^9^/L; platelet count, 214 × 10^9^/L; C-reactive protein level, 4.68 mg/L; procalcitonin level, 0.052 ng/mL; total bilirubin level, 12.1 μmol/L; alanine transaminase level, 6 U/L; aspartate aminotransferase level, 25 U/L; alkaline phosphatase level, 272 U/L; gamma-glutamyl transferase level, 169 U/L; and creatinine level, 37.0 μmol/L. The patient’s electrocardiogram revealed sinus tachycardia. Chest CT scans showed multiple plaques, and consolidation in both lungs and a typical fungal ball filling the left upper pulmonary cavity (Figures 1A, B).

**FIGURE 1:**
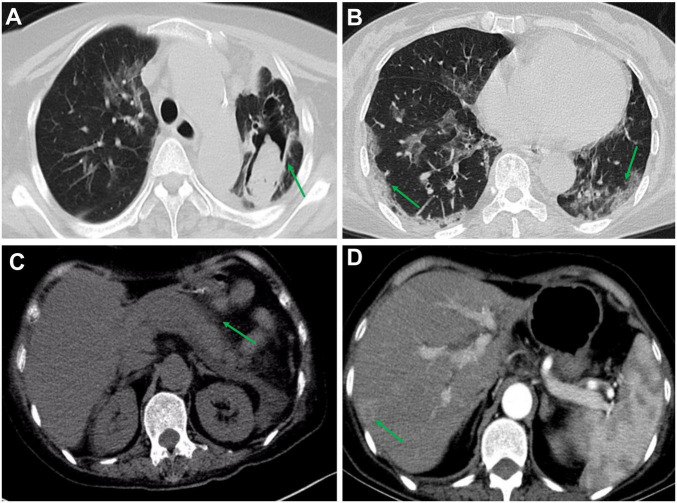
Diagnostic imaging findings. **(A)** Chest computed tomography (CT) showing a fungal ball (arrow) filling the left upper lobe cavity. **(B)** Chest CT demonstrating bilateral ground-glass opacities (arrow) consistent with COVID-19 pneumonia. **(C)** Abdominal CT revealing peripancreatic inflammatory changes surrounding the pancreatic body and tail, with no significant necrosis observed. **(D)** Enhanced CT of the abdomen disclosing a distinct nodular shadow within the right hepatic lobe.

The patient was started on oxygen inhalation, intravenous hemostatic agent therapy, and injections of voriconazole and piperacillin sodium and tazobactam sodium after admission. After 8 days without improvement, a fifth bronchial artery embolization was performed. Her vital signs remained largely stable, although her oxygen saturation was 94% by nasal cannula at 3 L/min. However, massive hemoptysis causing sudden suffocation occurred after the vascular intervention. She underwent immediate intubation and assisted breathing with invasive ventilation along with hemostasis, blood transfusion, continued antifungal treatment, anti-infection treatment, and nutritional support. Bronchoscopy revealed bloody purulent secretions. She was extubated successfully after 3 days and regained consciousness. However, she developed persistent fever, and her oxygen saturation level was 95%. Further, new moist rales were heard in the lower lobe of the right lung. Next-generation sequencing (NGS) of the sputum showed positive results for *Escherichia coli* (7 × 10^3^/mL), *Enterococcus faecium* (7 × 10^3^/mL), and 2019 novel coronavirus nucleic acid (4 × 10^3^/mL). The chest images showed a typical fungal ball filling the left upper pulmonary cavity and new ground shadows in both lungs. The patient received a 5-day oral course of Paxlovid (300 mg nirmatrelvir plus 100 mg ritonavir twice daily). Simultaneously, intravenous dexamethasone sodium phosphate was administered at 10 mg once daily for three consecutive days to mitigate the systemic inflammatory response, followed by a taper to 5 mg daily for an additional 2 days before discontinuation. Five days later, her fever resolved and her respiratory status improved. However, she developed upper abdominal tenderness. Her laboratory findings showed elevated serum amylase (503 U/L; reference range: 35–135 U/L) and serum lipase (353 U/L; reference range: 8–53 U/L), and abdominal CT showed peripancreatic inflammation (Figure 1C). Based on the 2013 Revised Atlanta Classification, the patient was diagnosed with mild acute pancreatitis (interstitial edematous pancreatitis), as there was no evidence of organ failure, local complications, or pancreatic necrosis on CT imaging (5). Common etiologies of acute pancreatitis were systematically excluded. Serum triglyceride and calcium levels were 0.7 mmol/L (reference range: <1.7 mmol/L) and 2.27 mmol/L (reference range: 2.11–2.52 mmol/L), respectively. The patient denied alcohol consumption, which was corroborated by family members. Abdominal CT demonstrated no evidence of cholelithiasis, biliary sludge, or bile duct dilatation. More advanced biliary imaging modalities, including magnetic resonance cholangiopancreatography (MRCP), endoscopic ultrasonography (EUS), and endoscopic retrograde cholangiopancreatography (ERCP), were either contraindicated or not feasible because of the patient’s recent massive hemoptysis, requirement for mechanical ventilation, and high bleeding risk. Consequently, the absence of more sensitive biliary imaging modalities (MRCP, EUS, and ERCP) means a microlithiasis or other occult biliary cause cannot be entirely ruled out. The patient was managed conservatively. A fasting regimen was initiated to reduce pancreatic stimulation, and continuous intravenous octreotide infusion (25 μg/h) was administered for 3 consecutive days to suppress pancreatic secretion. Fluid resuscitation with lactated Ringer’s solution was carefully titrated according to urine output and hemodynamic parameters to maintain adequate perfusion. Nutritional support was initially provided via total parenteral nutrition and subsequently transitioned to enteral feeding through a nasojejunal tube once abdominal tenderness subsided and serum amylase levels improved. These supportive measures, in combination with antiviral and anti-inflammatory therapies, contributed to clinical stabilization and recovery. At 12-month follow-up, the patient remained alive and clinically stable. However, recurrence of pulmonary tuberculosis and liver metastasis of GIST were confirmed (Figure 1D). Tuberculosis recurrence was diagnosed using sputum GeneXpert MTB/RIF testing, and anti-tuberculosis therapy was initiated, although treatment tolerance was poor and required dose adjustments. The patient continued to experience occasional hemoptysis and remained on voriconazole for chronic pulmonary aspergillosis. Notably, no late pancreatic complications were observed at follow-up, with no evidence of either exocrine or endocrine insufficiency.

Patient perspective: Written informed consent for publication was obtained. At the 12-month follow-up interview, the patient described the recurrent hemoptysis episodes as distressing, with the post-embolization suffocation episode being particularly traumatic. She expressed gratitude for the care received and acknowledged the complexity of her condition. She also reported difficulty distinguishing between respiratory and abdominal discomfort during the acute phase. At follow-up, she noted persistent fatigue and ongoing concern regarding the recurrence of tuberculosis and tumor progression, and emphasized the importance of coordinated multidisciplinary care in managing her condition.

## 3 Discussion

To our knowledge, this is the first reported case of life-threatening hemoptysis and acute pancreatitis temporally associated with COVID-19 in a patient with a complex medical history involving post-Whipple GIST, PTLD, and post-tuberculosis CCPA. This complexity posed significant diagnostic and therapeutic challenges.

Acute pancreatitis is defined as an inflammatory disease of the pancreas characterized by auto-digestion of pancreatic tissue by its own secretions. Infectious pathogens such as Coxsackievirus, mumps virus, and measles virus can also cause pancreatic impairment. Pancreatic involvement has also been reported in patients with COVID-19 after resolution of the viral infection (6). The development of AP in this patient presented a complex diagnostic challenge, as it coincided with the diagnosis of COVID-19 and the initiation of antiviral and anti-inflammatory therapy. Evidence suggests that SARS-CoV-2 may directly infect pancreatic acinar cells, which express high levels of the ACE2 receptor, or indirectly induce pancreatic injury through mechanisms such as systemic inflammatory response syndrome (SIRS), microcirculatory disturbances, and hypercoagulability, implicating COVID-19 as a potential significant contributor (3). In addition, drug-induced pancreatitis should be formally considered in the differential diagnosis. The patient was administered multiple medications, including Paxlovid (nirmatrelvir/ritonavir) and voriconazole. Ritonavir, as a potent CYP3A inhibitor, when co-administered with voriconazole – a substrate of CYP3A - may theoretically increase voriconazole exposure, thereby raising the risk of hepatotoxicity or other adverse effects. In this case, voriconazole was discontinued during Paxlovid therapy, and no significant hepatotoxicity was observed. Therapeutic drug monitoring confirmed that voriconazole serum concentrations remained within the therapeutic range over the entire course of voriconazole therapy. Regorafenib and imatinib had already been discontinued prior to hospital admission and were not considered active etiologic factors. Although drug-induced pancreatitis cannot be definitively verified or ruled out, the temporal association and potential pharmacological interactions indicate that drug-related mechanisms might have played a contributory role. In this case, common etiologies of acute pancreatitis, including gallstones, alcohol use, and hypertriglyceridemia, were excluded based on imaging and clinical evaluation. A comprehensive assessment of infectious, pharmacological, and surgical factors suggested that no single cause could be definitively identified. Instead, the pancreatitis was most likely multifactorial, resulting from the combined effects of SARS-CoV-2 infection, potential drug toxicity, altered postoperative anatomy, and the patient’s severe underlying comorbidities.

Despite surviving the acute phase, the patient developed TB recurrence and GIST metastasis at 1 year. The concurrent GIST and CCPA may have induced sustained immunosuppression, predisposing the patient to TB relapse and tumor progression. CCPA, a sequela of TB cavities, often requires long-term antifungals to control hemoptysis, but is limited by drug interactions, toxicity, and cost (7). Moreover, repeat embolization, while life-saving for massive hemoptysis, is palliative and carries risks. Thus, long-term monitoring for pancreatic insufficiency (exocrine/endocrine) after AP treatment and the Whipple procedure is essential.

The strengths of this report include its novelty as the first description of this unique combination of four coexisting conditions, the use of metagenomic next-generation sequencing (mNGS) for rapid pathogen identification, and the availability of comprehensive 12-month follow-up data. However, several limitations should be acknowledged. Firstly, advanced biliary imaging techniques, including MRCP, EUS, and ERCP, could not be performed due to the patient’s critical condition. Secondly, pancreatic biopsy or direct evidence of SARS-CoV-2 involvement in pancreatic tissue was not acquired. Thirdly, as a single-case report, the findings are not generalizable.

## 4 Conclusion

This case highlights the high risk of severe PTLD complications, such as CCPA, particularly in patients with a history of underlying malignancy. Furthermore, when such cases are further complicated by SARS-CoV-2 infection, clinicians should maintain heightened vigilance for both intrapulmonary and extrapulmonary manifestations, including pancreatic involvement. It is crucial to recognize that acute pancreatitis occurring during or after COVID-19 may demonstrate a temporal association; nevertheless, other potential etiologies must be thoroughly investigated and excluded. Early recognition and multidisciplinary management are key to improving outcomes in complex, multi-morbid cases.

## Data Availability

The raw data supporting the conclusions of this article will be made available by the authors, without undue reservation.
